# Implementation of HLA-B*15:02 Genotyping as Standard-of-Care for Reducing Carbamazepine/Oxcarbazepine Induced Cutaneous Adverse Drug Reactions in Thailand

**DOI:** 10.3389/fphar.2022.867490

**Published:** 2022-07-05

**Authors:** Kanyawan Tiwattanon, Shobana John, Napatrupron Koomdee, Pimonpan Jinda, Jiratha Rachanakul, Thawinee Jantararoungtong, Nutthan Nuntharadthanaphong, Chiraphat Kloypan, Mohitosh Biswas, Apisit Boongird, Chonlaphat Sukasem

**Affiliations:** ^1^ Division of Neurology, Department of Medicine, Faculty of Medicine Ramathibodi Hospital Mahidol University, Bangkok, Thailand; ^2^ Division of Pharmacogenomics and Personalized Medicine, Department of Pathology, Faculty of Medicine Ramathibodi Hospital, Mahidol University, Bangkok, Thailand; ^3^ Laboratory for Pharmacogenomics, Somdech Phra Debaratana Medical Center (SDMC), Ramathibodi Hospital, Bangkok, Thailand; ^4^ Unit of Excellence in Integrative Molecular Biomedicine, School of Allied Health Sciences, University of Phayao, Phayao, Thailand; ^5^ Division of Clinical Immunology and Transfusion Science, Department of Medical Technology, School of Allied Health Sciences, University of Phayao, Phayao, Thailand; ^6^ Department of Pharmacy, University of Rajshahi, Rajshahi, Bangladesh; ^7^ Ramathibodi Multidisciplinary Center (RMEC), Faculty of Medicine Ramathibodi Hospital, Mahidol University, Bangkok, Thailand; ^8^ Pharmacogenomics and Precision Medicine Clinic, The Preventive Genomics and Family Check-up Services Center, Bumrungrad International Hospital, Bangkok, Thailand; ^9^ MRC Centre for Drug Safety Science, Department of Pharmacology and Therapeutics, Institute of Systems, Molecular and Integrative Biology, University of Liverpool, Liverpool, United Kingdom

**Keywords:** carbamazepine, *HLA-B* risk alleles, pharmacogenomics, cutaneous adverse drug reactions, precision medicine

## Abstract

**Objective:** This study aimed to investigate the clinical impact of HLA-B*15:02 pharmacogenomics (PGx) testing before carbamazepine (CBZ)/oxcarbazepine (OXC) prescriptions and to determine whether this PGx testing was associated with the reduction of CBZ/OXC-induced cutaneous adverse drug reactions (CADRs) in Thailand.

**Methods:** This retrospective observational cohort study was conducted by obtaining relevant HLA-B*15:02 PGx-testing and clinical data from electronic medical records during 2011–2020. 384 patient data were included in this study to investigate the clinical decision on CBZ/OXC usage before and after the HLA-B*15:02 PGx testing, and 1,539 patient data were included in this study to demonstrate the incidence of CBZ/OXC-induced SCARs and SJS between HLA-B*15:02 tested and non-tested patients. To analyze and summarize the results, descriptive statistics were employed, and Fisher exact test was used to compare the clinical difference between the HLA-B*15:02 positive and negative groups and to compare the differences of SCARs incidence.

**Results:** 384 patients were included in this study as per the inclusion criteria. Of these, 70 patients carried HLA-B*15:02, of which 63 and 65 patients were not prescribed with CBZ/OXC before and after the availability of genotyping results, respectively. In the remaining HLA-B*15:02 non-carriers, 48, and 189 patients were prescribed CBZ/OXC before and after genotyping results were available, respectively. The findings of this study showed that the incidence of SCARs of CBZ/OXC was significantly lower (*p* < 0.001) in the HLA-B*15:02 screening arm than in the non-screening arm.

**Conclusion:** *HLA-B* pharmacogenetics testing influenced the selection of appropriate AEDs. The presence of mild rash in the HLA-B*15:02 negative group indicates that other genetic biomarker (HLA-A*31:01) and/or non-genetic variables are involved in CBZ/OXC-induced CADRs, emphasizing that CBZ/OXC prescriptions necessitate CADR monitoring. The hospital policy and clinical decision support (CDS) alert system is essential to overcome the barriers associated with the utilization of PGx guidelines into clinical practice.

## Introduction

Carbamazepine (CBZ) is a first-generation antiepileptic drugs (AEDs) used to treat a variety of neurological and psychiatric problems, including epilepsy, trigeminal neuralgia, and bipolar disorders. In Thais, there has been a well-documented association between HLA-B*15:02 and CBZ-induced Stevens-Johnson syndrome (SJS) and toxic epidermal necrolysis (TEN) (Hung et al., 2006; Locharernkul et al., 2008; Tassaneeyakul et al., 2010; Tangamornsuksan et al., 2013; Sukasem et al., 2018; Sukasem et al., 2021b). In 2013, the government launched a policy requiring HLA-B*15:02 pharmacogenetic (PGx) testing before the start of CBZ in Bangkok as a pilot study (Department of Medical Sciences, 2013). Subsequently, national policy screening for HLA-B*15:02 was reinforced by the National Health Security Office (NHSO) in 2018 throughout Thailand (Ang et al., 2017; Chang et al., 2020; Sukasem et al., 2021a; Jantararoungtong et al., 2021). Besides HLA-B*15:02, some other variants such as HLA-B*15:08, HLA-B*15:11 and HLA-B*15:21 may also affect the safety of CBZ and found to be other *HLA-B* risk *alleles* associated with CBZ-induced cutaneous adverse drug reactions (CADRs) (Jaruthamsophon et al., 2017; Volpi et al., 2018; Kloypan et al., 2021).

In addition, Chen et al. reported a strong association between the HLA-B*15:02 allele and oxcarbazepine (OXC)-induced SJS/TEN in Chinese and Thai populations (Chen et al., 2017). Consequently, the Clinical Pharmacogenetics Implementation Consortium (CPIC) guideline recommended to avoid the prescription of CBZ and OXC in HLA-B*15:02 carrier (Phillips et al., 2018).

Although knowledge and research on PGx testing in Thailand have been rapidly expanding in recent years (Jaruthamsophon et al., 2017), there has been no longitudinal research on the application of HLA-B*15:02 genotype results in daily clinical practice, including documentation of severe cutaneous adverse drug reactions (SCARs) and new adverse drug reactions (ADRs) in electronic medical records, patterns of AEDs prescriptions based on genotype results, and assessment of the CADRs. This is the first research of its kind in Thailand, and it focuses on these fascinating topics.

The goal of this study was to investigate how PGx testing (HLA-B*15:02) affected the CBZ/OXC prescriptions and the reduction of CBZ/OXC-induced SCARs in Thais. The findings of this study have the potential to help physicians regarding the prescription of appropriate AEDs in clinical practice for those patients carrying HLA-B*15:02 CBZ/OXC-risk alleles and to support NHSO Thailand’s HLA-B*15:02 screening as the national policy.

## Methodology

### Study Population

**FIGURE 1:**
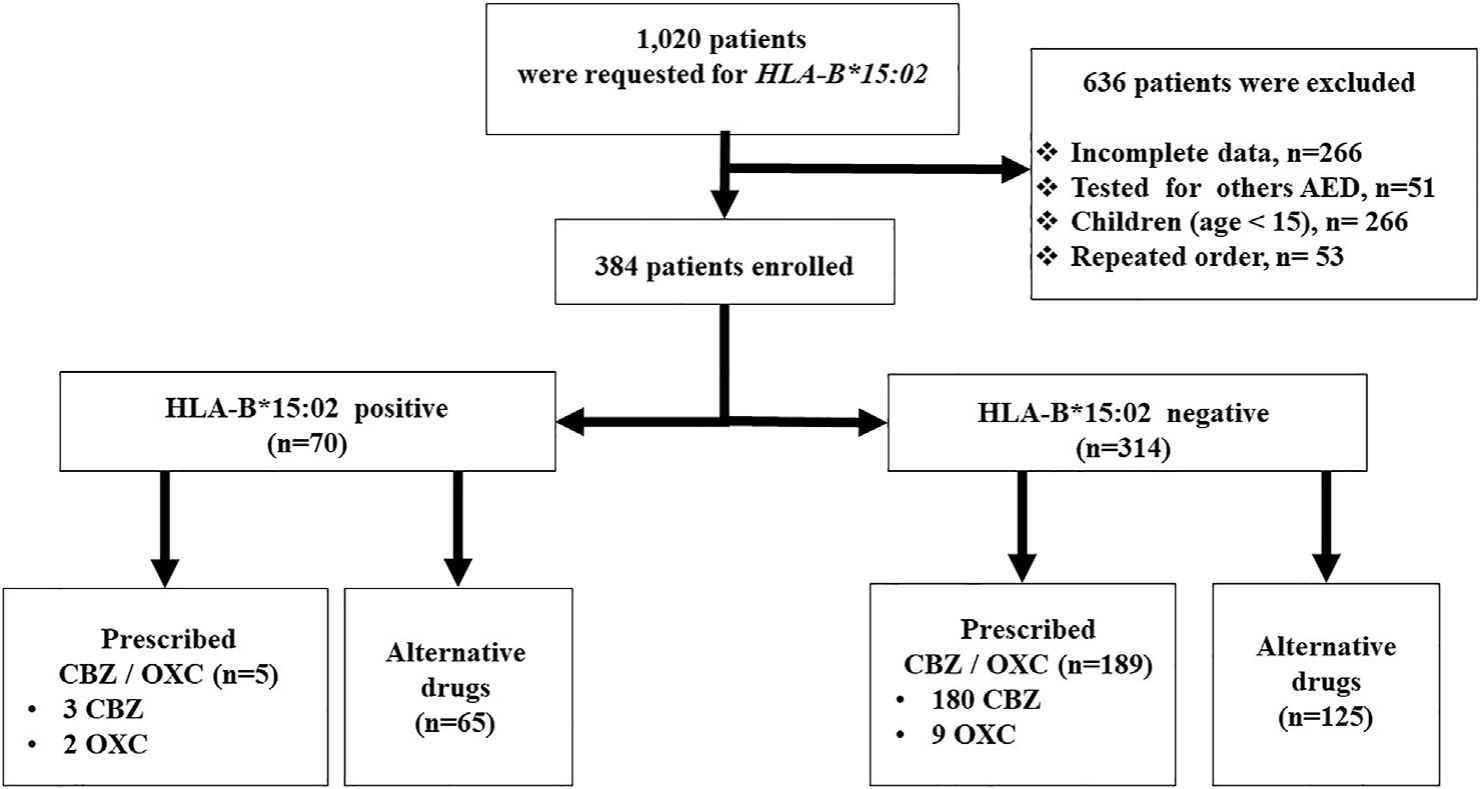
The flowchart of patients recruitment and clinical decision in HLA-B*15:02 positive (n = 70) and HLA-B*15:02 negative (n = 314) in Ramathibodi Hospital, Bangkok.

This was a retrospective cohort study conducted at the Ramathibodi Hospital of the Faculty of Medicine. In order to determine the influence of HLA-B*15:02 PGx testing in clinical practice, 1,020 patients with performed HLA-B*15:02 PGx testing between 2011 and 2020 were included. Among 1,020 patients, 636 patients were excluded due to incomplete data (n = 266), PGx tested for other AEDs (n = 51), repeated PGx order (n = 53), and less than 15 years of age (n = 266) (Figure 1). This study did not include *HLA-B* genotyping that was performed to test the association of drug induced CADRs. Subsequently, 384 patients were enrolled for analysis. Patients’ demographic information, such as age, gender, diagnosis, and drug-related information, such as the number of CBZ prescriptions and their association to HLA-B*15:02 genotyping results, CADRs types, drug allergic history, PGx testing, number of comorbidities, types of comorbidities, and alternative drugs utilized were recorded. All CADRs were diagnosed and confirmed by a dermatologist at Ramathibodi Hospital in Thailand.

To demonstrate how prospective HLA-B*15:02 testing likely reduces the occurrence of hypersensitivity reactions to CBZ/OXC, we recruited 1,539 patients data who were prescribed CBZ/OXC for a period of 2013–2021 and further classified them as tested for HLA-B*15:02 group or not tested for HLA-B*15:02 group. We retrospectively collected CBZ/OXC prescribing data, HLA-B*15:02 testing data, and SCARs occurrence data for this part of the study.

**FIGURE 2:**
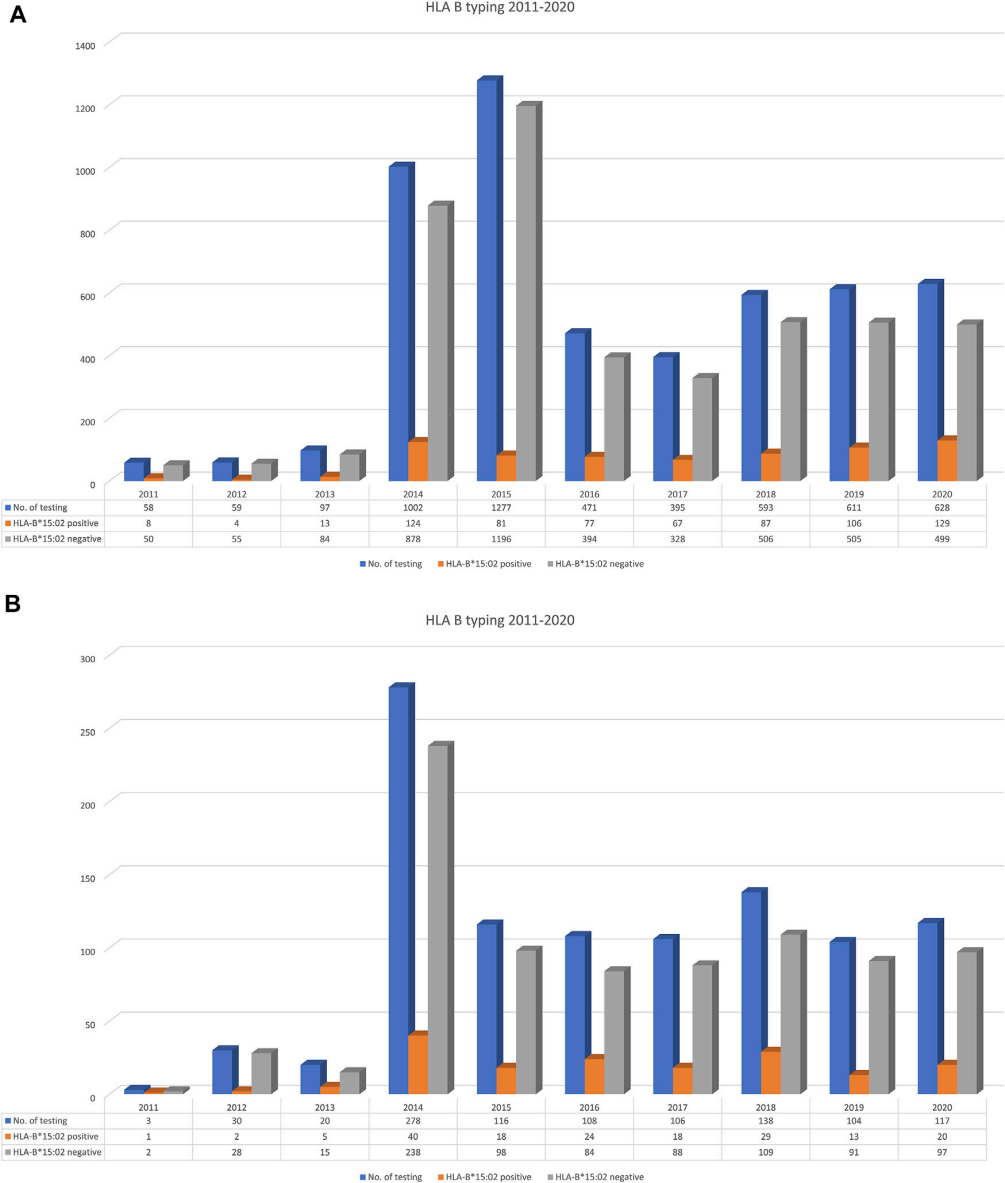
Trend of HLA-B*15:02 testing requested by out-side [n = 5,191; **(A)**] and in-side [n = 1,020; **(B)**] of Ramathibodi Hospital (19.64%) during 2011–2020.

To exemplify the overall trend of HLA-B*15:02 testing requests within and outside of Ramathibodi Hospital, we collected overall HLA-B*15:02 PGx testing for CBZ/OXC that was performed during 2011–2020 and presented them in percentage as per year (Figure 2).

The studies involving human participants were reviewed and approved by the Ethical Review Committee on Research Involving Human Subjects, Faculty of Medicine Ramathibodi Hospital, Mahidol University. The patients provided written informed consent to participate in this study.

### *HLA-B* genotyping

Genomic DNA samples were isolated from EDTA blood, using the MagNAprue Compact Nucleic Acid Isolation kits (Roche Applied Science, Mannheim, Germany). The quality of genomic DNA was measured by NanoDrop^®^ ND-1000 (Thermo Scientific, Wilmington, United States). *HLA-B* alleles were analyzed by the polymerase chain reaction-sequence specific oligonucleotide probe (PCR-SSOP) assay and Luminex™ Multiplex Technology with well established protocols.

In brief, the DNA sample obtained from patients was amplified by polymerase chain reaction (PCR). The PCR product was then hybridized against a panel of sequence specific oligonucleotide probes on coated polystyrene microspheres that had sequences complementary to stretches of polymorphism within the target *HLA-B.* The amplicon-probe complex was then visualized using a colorimetric reaction and fluorescence detection technology by the Luminex®IS 100 system (Luminex Corporation, Austin, Texas, United States). Analysis of the *HLA* class I alleles were performed using HLA fusion software version 2.0 (One Lambda, Canoga Park, CA, United States). For each allele, the results were reported as either HLA-B*15:02 positive or negative.

### Data Analysis

Descriptive statistics were employed to summarize and analyze the collected data. The Fisher exact test was used to compare the clinical characteristics of the HLA-B*15:02 positive (positive group) and HLA-B*15:02 negative (negative group) and to calculate the difference of the SCARs incidence between the HLA-B*15:02 tested group and non-tested groups.

## Results

### Patients Characteristics, Diagnosis, Comorbidities, Adverse Drug Reactions (ADRs), Drug Allergy

Out of 1,020 patients undertaking HLA-B*15:02 genotyping tests during 2011–2020, only 384 patients were included as per the inclusion criteria in this study (Figure 1). Incomplete data (n = 266), tested for other AEDs (n = 51), Patients’ data under the age of 15 (n = 266), and repeatedly requested data (n = 53) were excluded from this study. Our study population consists of 243 (63.28%) females and 141 (36.72%) males. The age range of this population was 15–95, with a mean age of 49.23 ± 19.79 (SD). Out of 384 patients, 112 (29.16%) were diagnosed with different types of epilepsy. The second most common disease for the CBZ indication was trigeminal neuralgia (n = 95, 24.74%). Neuropathic pain (n = 64, 16.67%), hemifacial spasm (n = 14, 3.65%), tonic spasm (n = 13, 3.39%), and bipolar disorder (n = 23, 5.99%) were the other commonly diagnosed diseases for which CBZ was prescribed in this study. Sixty-three cases were in miscellaneous categories (Table 1).

**TABLE 1:** Patients characteristics, diagnosis, comorbidities, adverse drug reactions (ADRs), drug allergy.

Parameter	Total	HLA-B*15:02 Positive	HLA-B*15:02 Negative	p-value^a^
Number of Patients (%)	384	70 (18.23)	314 (81.77)	
Age (year)				
Range	15–95	15–94	15–95	-
Mean±SD	49.23 ± 19.79	46.34 ± 20.25	49.89 ± 19.66	-
Gender (%)				
Male	141 (36.72)	32 (45.71)	109 (34.71)	<0.0001^c^
Female	243 (63.28)	38 (54.29)	205 (62.29)	
Diagnosis (%)				
Epilepsy	112 (29.16)	20 (28.57)	92 (29.30)	-
Trigeminal Neuralgia	95 (24.74)	18 (25.71)	77 (24.52)	-
Neuropathic pain	64 (16.67)	14 (20.0)	50 (15.92)	-
Hemifacial spasm	14 (3.65)	0	14 (4.46)	-
Tonic spasm	13 (3.39)	1 (1.43)	12 (3.82)	-
Bipolar disorder	23 (5.99)	4 (5.71)	19 (6.05)	-
Miscellaneous	63 (16.40)	13 (18.57)	50 (15.92)	-
CBZ/OXC Dosage (mg)	CBZ50-800/OXC300-600	CBZ200-800/OXC300-600	CBZ 50–800/OXC300-600	-
ADR History (%)	140 (36.46)	33 (47.14)	107 (34.08)	0.05
CADRs and Non CADRs (%)				
CADRs	71 (18.49)	18 (25.71)	53 (16.88)	0.09
Non-CADRs	69 (17.97)	15 (21.43)	54 (17.20)	-
CBZ/OXC prescription (%)				
Prescribed	194 (50.52)	5 (7.14)	189 (60.19)	<0.0001
Not prescribed	190 (49.48)	65 (92.86)	125 (39.81)	-
Comorbidities (%)	108 (28.12)	17 (24.29)	91 (28.98)	0.46
Types of Comorbidities (%)				
SLE	9 (2.34)	3 (4.29)	6 (1.91)	-
RA	3 (0.78)	2 (2.86)	1 (0.31)	-
CA	15 (3.90)	3 (4.29)	12 (3.82)	-
HIV	4 (1.04)	1 (1.43)	3 (0.96)	-
DM	9 (2.34)	2 (2.86)	7 (2.23)	-
CNS infections	6 (1.56)	1 (1.43)	5 (1.59)	-
Thyroid	6 (1.56)	1 (1.43)	5 (1.59)	-
Others	56 (14.58)	4 (5.71)	52 (16.56)	-
Past Drug Allergy (%)	140 (36.46)	33 (47.14)	107 (34.08)	0.05
Other AEDs	48 (12.50)	13 (18.57)	35 (11.15)	-
Other Drugs	92 (23.96)	20 (28.57)	72 (22.93)	-
Past PGx testing (%)	40 (10.42)	4 (5.71)	36 (11.46)	0.06
B*13:01	14 (3.65)	1 (1.43)	13 (4.14)	-
B*57:01	2 (0.52)	1 (1.43)	1^b^	-
B*58:01	16 (4.17)	1 (1.43)	15 (4.78)	-
B*35:05	9 (2.34)	1 (1.43)	8 (2.55)	-
Number of CADRs in CBZ/OXC used (%)	21 (5.47)	0	21 (6.69)	0.01
Mild	19 (4.95)	0	19 (6.05)	-
Severe	2 (0.52)	0	2 (0.64)	-
Number of non-CADRs in CBZ/OXC used (%)	22 (5.73)	0	22 (7.01)	0.01

CBZ, Carbamazepine; OXC, Oxcarbazepine; RA, Rheumatoid arthritis; HIV, Human immunodeficiency virus; SLE, Systemic Lupus Erythromatous; CA, Carcinoma; DM, Diabetes Mellitus; ADR, Adverse drug reactions; CADRS, Cutaneous adverse drug reactions; AEDs, Antiepileptic Drugs; PGx, Pharmacogenetic Testing; SD, Standard Deviation.

a p value calculated using fisher exact test

b one patient had 2 PGx testing history

c p value calculated between male and female.

Comorbidities were discovered in 108 (28.12%) patients, 17 (17/70, 24.29%) of them were HLA-B*15:02 positive and the remaining 91 (91/314, 28.98%) were HLA-B*15:02 negative. The CBZ was prescribed in doses ranging from 50 to 800 mg, while the OXC was prescribed in doses ranging from 300 to 600 mg.

According to the findings, 36.46% (n = 140) of the patients had previous ADR history, while 18.49% (n = 71) had previously experienced CADRs. HLA-B*15:02 positive patients had higher CADRs than HLA-B*15:02 negative patients (18/70, 25.71% vs 53/314, 16.88%, *p* = 0.09), however, the difference was insignificant. 48 of the 384 patients had a prior history of AED allergy, with 18.57% (13/70) and 11.15% (35/314) belonging to the HLA-B*15:02 positive and negative groups, respectively (*p* = 0.05).

### Clinical Decision on CBZ/OXC Usage Before and After the HLA-B*15:02 PGx Testing

Table 2 shows the status of a clinical decision on CBZ/OXC prescription. Because all of the patients were scheduled to receive CBZ/OXC therapy, HLA-B*15:02 testing was performed as per the national policy. Out of 384 patients, the clinical decision on CBZ/OXC was put on hold for 85.67% of patients and requested the HLA-B*15:02 testing prior prescription. Because of the clinical urgency, 55 patients were prescribed CBZ/OXC while the HLA-B*15:02 testing was ordered. HLA-B*15:02 carriers were found in 70 of the 384 cases. This accounts for 18.23% of the overall study population. Fortunately, CBZ was not prescribed in 63 of 70 patients with HLA-B*15:02 carriers before the genotyping results were available; and after receiving the HLA-B*15:02 results, CBZ/OXC was not prescribed in 65 of 70 patients. Only 48 patients in the negative group received CBZ/OXC before the HLA-B*15:02 requested, but 189 patients with HLA-B*15:02 negative were prescribed CBZ/OXC following the results available in the electronic medical record (EMRs) (Table 2). In this cohort, five patients were remain treated with CBZ/OXC-based regimen since they were received this regimen for longer than 3 months and had no CADRs. On the other hand, 189 patients in HLA-B*15:02 negative group were administered CBZ/OXC as recommended. Surprisingly, 21 patients with negative HLA-B*15:02 had CADRs, of which 2 patients (9.52%) were SCARs (one DRESS and one SJS cases) and 19 patients (90.48%) were mild rash. Non-CADRs were also observed in 22 patients with negative HLA-B*15:02, with dizziness being the most common ADRs (Table 1).

**TABLE 2:** Clinical decision on CBZ/OXC usage before and after received the HLA-B*15:02 PGx testing.

Therapeutics	Before the HLA-B*15:02 Testing				After Received the HLA-B*15:02 reports		
	+ve (n = 70)	-ve (n = 314)	Total (n = 384)	Hold CD	+ve (n = 70)	-ve (n = 314)	Total (n = 384)
CBZ/OXC	7 (10.00%)	48 (15.29%)	55 (14.32%)	329 (85.67)	5 (7.14%)	189 (60.19%)	194 (50.52%)
Alternative drugs	63 (90.00%)	266 (84.71%)	329 (85.68%)	-	65 (92.86%)	125 (39.80%)	190 (49.48%)

CD, Clinical Decision; CBZ, Carbamazepine; OXC, Oxcarbazepine; +ve, HLA-B*15:02 positive; -ve, HLA-B*15:02 negative.

### Cases of Carbamazepine-Induced SCARs Without PGx Testing

In this study, 13 cases were reported as CBZ-induced SCARs after received CBZ without PGx testing during 2013–2021 (Table 3). Among them, the patients were diagnosed as SJS/TEN (n = 10), Drug rash with eosinophilia and systemic symptoms (DRESS, n = 2) and fix-drug eruption (FDE, n = 1). Remarkably, HLA-B*15:02 were positive in all SJS/TEN cases (10/10, 100%), where as negative in DRESS and FDE cases. Two patients with CBZ-induced DRESS showed no HLA-B*15:02 positive (HLA-B*27:06/58:01 and HLA-B*40:01/58:01)*.*

**TABLE 3:** Cases of CBZ/OXC-induced SCARs and fixed drug eruption after received CBZ without PGx testing.

Case	Gender	Age	*HLA-B* Genotype	ADRs type	Year of Report
1	F	51	B*15:02/46:01	SJS/TEN	2013
2	F	32	B*15:02/44:03	SJS/TEN	2014
3	M	93	B*40:01/58:01	DRESS	2015
4	M	35	B*15:02/39:09	SJS	2015
5	M	64	B*15:02/58:01	SJS	2015
6	F	38	B*27:06/58:01	DRESS	2016
7	F	40	B*15:02/18:01	SJS	2016
8	F	43	B*15:02/46:01	SJS	2016
9	F	19	B*15:02/15:02	SJS/TEN	2017
10	M	23	B*15:02/15:25	SJS	2018
11	M	54	B*15:02/40:01	TEN	2018
12	F	57	B*15:02/46:01	TEN	2019
13	F	56	*DNA not available*	FDE	2019

ADRs, Adverse drug reactions; CBZ, carbamazepine; DRESS, drug reaction with eosinophilia and systemic symptoms; FDE, fixed drug eruption; SCARs, Severe cutaneous adverse drug reactions; SJS, Stevens-Johnson syndrome; TEN, toxic epidermal necrolysis.

### Incidence of SJS-TEN Between Tested and Non-tested Patients Who Prescribed With CBZ/OXC

Available data demonstrated that prospective HLA-B*15:02 probably reduces the incidence of hypersensitivity reactions to CBZ/OXC (Table 4). Of the 1,539 patients who were prescribed for CBZ/OXC, 916 (59.5%) were tested for the HLA-B*15:02 allele and 623 (40.5%) were not tested. The incidence of clinically diagnosed SCARs to CBZ/OXC was significantly lower (*p* < 0.001) in the screening arm (0.22%) when compared to the non-screening arm (1.93%). Moreover, 10 of 623 (1.61%) of non-tested patients suffered from SJS/TEN, while only 1 of 916 (0.22%) was observed in tested patients (*p* < 0.001).

**TABLE 4:** Incidence of CBZ/OXC-induced SCAR and SJS between HLA-B*15:02 tested patients and non-tested patients.

Variable	Prescripted with CBZ/OXC	No HLA-B*15:02 Testing	Testing for HLA-B*15:02	P-Value^a^
Number of Patients (%)	1,539	623 (40.5)	916 (59.5)	-
CBZ/OXC-induced SCARs (no.)	14	12	2	0.0001
Incidence of CBZ/OXC-induced SCARs (%)	0.91	1.93	0.22	-
CBZ/OXC-induced SJS-TEN (no.)	11	10	1	0.0003
Incidence of CBZ/OXC-induced SJS-TEN (%)	0.71	1.61	0.11	-

a p value (<0.05) calculated using fisher exact test between tested and non-tested patients.

### Trend of HLA-B*15:02 Testing Requested by Out-Side and In-Side of Ramathibodi Hospital

The Laboratory for Pharmacogenomics (PPM), Ramathibodi Hospital has been established as the first reference laboratory for PGx testing in Thailand. Figure 2 illustrates the trend of HLA-B*15:02 testing in the country and also hospital during 2011–2020. Totally, 5,191 PGx testing for CBZ/OXC were performed during 2011–2020 in Thailand whereas 1,020 (19.64%) patients were requested HLA-B*15:02 for CBZ/OXC treatment in Ramathibodi Hospital. Interestingly, only 58 samples were requested by clinicians in 2011, but the number of PGx testing was escalated to 1,002 and 1,277 in 2014 and 2015, respectively. The similar trend was found in Ramathibodi Hospital; it started with three patients in 2011 and has gradually risen in 2014 (n = 278) and dropped in 2015 (n = 116), 2016 (n = 108), 2017 (n = 106), 2018 (n = 138), 2019 (n = 104) and 2020 (n = 117). Totally, 696 (13.27%) and 170 (16.67%)HLA-B*15:02 carriers were detected in Thailand and Ramathibodi Hospital, respectively.

### Alternative Drugs Prescription in Patients With HLA-B*15:02 Positive

**FIGURE 3:**
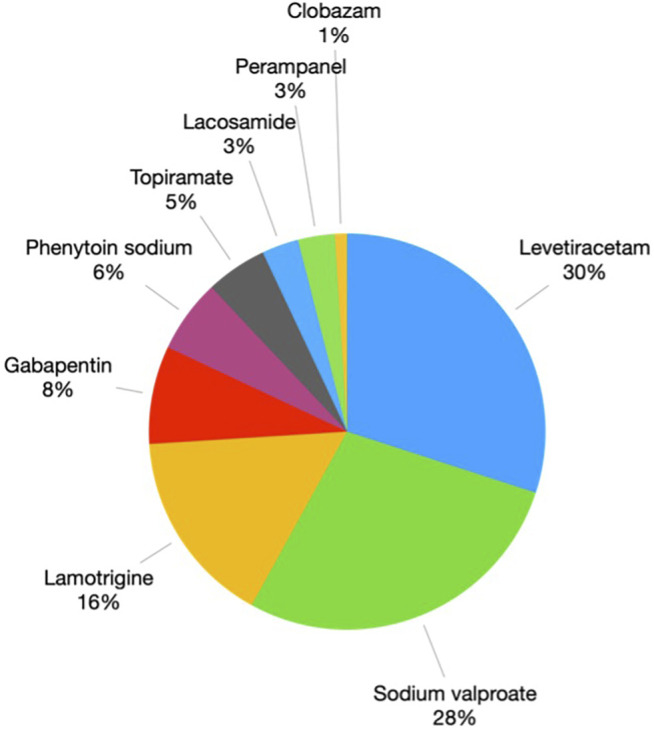
Alternative drugs were prescribed in patients with HLA-B*15:02 positive.

In order to prevent the SJS/TEN from CBZ/OXC, the alternative AEDs choices were prescribed in HLA-B*15:02 carriers as per the Thailand Clinical Practice Guidelines for Epilepsy and also NHSO policies. Figure 3 depicts the alternative medications that were utilised in place of CBZ/OXC. The most widely utilised alternative AEDs were levetiracetam (30%), valproate (28%) and lamotrigine (LTG) (16%).

## Discussion

This is the first retrospective cohort study in Thailand to determine the clinical impact of pharmacogenomics (PGx) testing on carbamazepine/oxcarbazepine (CBZ/OXC) prescribing. The female preponderance was seen in the PGx testing population in this study, and it may be because of the higher incidence of hypersensitivity reactions among females (Saksit et al., 2017). Many earlier research studies support this fact. For example, a study conducted by Alvestad et al. (2007) reported that a striking difference in the incidence of hypersensitivity was seen between males (8%) and females (19%) with *p* < 0.001. This association is highly dependent on the age group; reproductive years 20–50 are the most significant age group. The explanation for this is that sex hormones alter the immunological process of rash. Androgens limit the inflammatory response more than endogenous glucocorticoids, whereas female sex hormones enhance the immunological response both pathologically and physiologically (Talal, 1987; Da Silva, 1999; Osman, 2003).

Pharmacogenomic testing for HLA-B*15:02 is now standard practice in Thailand. In this study, physicians requested the PGx testing for CBZ/OXC and hold the prescription until received the PGx report for 85.67% of 384 patients. This was complying with Clinical Practice Guidelines for Epilepsy (2018) of Epilepsy Society of Thailand and the Clinical Pharmacogenetics Implementation Consortium Guideline for HLA Genotype and Use of Carbamazepine and Oxcarbazepine: 2017 Update (Epilepsy Society of Thailand, 2018; Phillips et al., 2018). Do not use CBZ in naïve-patients that are positive for HLA-B*15:02. If patient used CBZ for longer than 3 months without incidence of adverse reactions, consider use with caution (Phillips et al., 2018). However, seven patients were found to receive CBZ/OXC while requesting the HLA-B*15:02 PGx testing. Two of them were CBZ naïve-patients and prescribed with CBZ due to clinical urgency and five were identified as CBZ/OXC-treated patients (used CBZ/OXC for longer than 3 months). Therefore, alternative drugs were prescribed in 65 (92.86%) patients who carried HLA-B*15:02 (positive group).

Among the 314, negative HLA-B*15:02 group, 60% were prescribed with CBZ/OXC, whereas the remaining 40% switched over to other AEDs overall before and after the PGx test. The retrospective follow up analysis showed that lengthy *HLA-B* testing results turnaround time and/or lengthy reach out time of the available PGx testing results to physicians were the two important reasons for it. This is a very common barrier while translating the PGx into the real clinical world (Krebs and Milani, 2019). Thailand has recently seen a lot of technological improvement in genetic analysis but we used samples from 2011 in our research. This could explain why clinician prescribed CBZ/OXC without waiting for the PGx results in 55 (14.32%) patients. Physicians, on the other hand, awaited for the PGx results of 329 patients before making a clinical decision. This supports our hypothesis that physician CBZ/OXC prescribing decisions were influenced by HLA-B*15:02 genotyping. Moreover, 92.85% (65/70) of HLA-B*15:02 risk allele carriers were avoided by CBZ/OXC, so it can be assumed that the risk population is separated and SCARs are prevented in this population.

However, the screening for HLA-B*15:02 is not a compulsory testing in Thailand prior to initiation of CBZ treatment. During 2013–2019, CBZ-induced SCARs were reported from 13 patients who received CBZ without PGx screening in Thai-SCARs cohort. Remarkably, HLA-B*15:02 were positive in all SJS/TEN cases (n = 10, 100%), where as negative HLA-B*15:02 in DRESS and FDE. Assumably, these patients can be prevented from SJS/TEN if the HLA-B*15:02 PGx testing was required as a mandatory testing for CBZ prescription. Additionally, the hospital policy and clinical decision support (CDS) alert system is essential to overcome the barriers associated with the utilization of PGx guidelines into clinical practice. The PGx-CDS should be integrated into EMRs and pop-up alert in the therapeutics order system recommending the clinician to order a PGx screening test prior initiation of preventable drug-induced SCARs such as CBZ/OXC (HLA-B*15:02), allopurinol (HLA-B*58:01), abacavir (HLA-B*57:01) and cotrimoxazole/dapsone (HLA-B*13:01) (Sukasem et al., 2016; Sukasem et al., 2020; Kloypan et al., 2021; Satapornpong et al., 2021).

An earlier study in Thailand reported that out of 214 SCARs-treated patients over a period of 10 years, 23 patients (10.6%) were treated with CBZ, and this shows that CBZ is the major culprit for SCARs. If MPE and other mild-moderate level reactions were included, this number could go higher than this. However, it cannot be assumed that all the risk allele carriers will have CBZ-induced CADRs. In this study, 7.14% of patients who carried HLA-B*15:02 allele did not manifest any CADRs. Because the frequency of SJS/TEN is substantially lower than the frequency of the risk allele in these groups, previous studies have shown that the test’s positive predictive value is poor (1–5%). Although having HLA-B*15:02 increases the risk of CBZ/OXC induced SJS/TEN by up to >700-fold compared to non-carriers, the vast majority of patients with HLA-B*15:02 (95%–99%) do not develop SJS/TEN from CBZ/OXC (Amstutz et al., 2014).

However, in this study if the mild reactions are included this rate goes up to 11%. The reason could be that 29% of the negative patients were reported with multiple comorbidities and most of them were immune related disorders like Systemic lupus erythematosus (SLE), hypo/hyperthyroid, cancer, rheumatoid arthritis (RA), diabetes mellitus (DM), hypertension (HTN), and central nervous system (CNS) infections. Earlier studies showed that some non-clinical risk factors may be involved in the initiation of CADRs (Campos-Fernández Mdel et al., 2005; Patel et al., 2014; John et al., 2021).

Unexpectedly, 2 cases with CBZ-induced SCARs (DRESS and SJS) were reported among 189 patients with HLA-B*15:02 negative after CBZ/OXC treated patients. Likewise, 2 cases with CBZ-induced DRESS were reported after CBZ treatment without HLA-B*15:02 PGx testing. Commonly, the HLA-B*15:02 allele has been reported to be specifically associated with CBZ-induced SJS/TEN in Asian populations, and no associations have been reported for drug-induced MPE and DRESS (phenotype-specific biomarker) (Sukasem et al., 2021b). In contrast, HLA-A*31:01 was reported to be associated with CBZ-induced DRESS and MPE (Mockenhaupt et al., 2019; Ahmed et al., 2021). US-FDA recommended that HLA-B*15:02 and HLA-A*31:01 genotypes should be detected to stratify the high risk patients prior CBZ prescription. The use of CBZ should be avoided in patients who test positive for the HLA-A*31:01 or HLA-B*15:02 alleles (US Food and Drug Administration, 2009). However, conflicting evidence has been found in this study, one SJS patient was reported from negative of HLA-B*15:02 group. This suggested that other genetic and clinical factors may also influence a patient’s risk for adverse reactions.

PGx testing has been implemented in many hospitals throughout the world over the last decade to guarantee that the best treatment is chosen. For example, 27 institutions in the United States have established numerous PGx implementation programmes (Dunnenberger et al., 2015; Volpi et al., 2018), while other PGx implementation programmes in Europe and East Asia include EU-funded Ubiquitous Pharmacogenomics (U-PGx), PREemptive Pharmacogenomic testing for the prevention of Adverse Drug Reactions (PREPARE) (van der Wouden et al., 2017), and the Southeast Asian Pharmacogenomics Research Network (SEAPharm) (RIKEN, 2012; Chumnumwat et al., 2019).

We discovered that the number of HLA-B*15:02 tests per year has increased significantly since 2013 in Thailand. The reason for the high PGx testing could be because of the implementation of a pilot project for HLA-B*15:02 PGx testing before prescribing CBZ in Bangkok by the Department of Medical Sciences, Ministry of Public Health, Thailand. This phenomenon was emphasised later by NHSO announcement for implementation of national screening policy in 2018.

In the HLA-B*15:02 positive group, levetiracetam, sodium valproate, LTG, gabapentin, and phenytoin (PHT) sodium were the most prescribed as alternative AEDs in epilepsy. The reason that levetiracetam and sodium valproate were used is that they are non-aromatic AEDs with a low rate of cross-reactivity with CADRs. In 2014, Li et al. (2015) reported a significant association between HLA-B*15:02 and PHT or LTG-induced SJS/TEN. Nevertheless, our study found that LTG and PHT were prescribed in HLA-B*15:02 positive patients without any ADRs. The reason could be the strength of the association is much weaker than it was for CBZ/OXC-related SJS/TEN. A study from Thailand reported a moderate association (limited evidence) (Locharernkul et al., 2008) and a case report of Han Chinese also showed a weak association (insufficient evidence) (Shi et al., 2012).

Although the retrospective nature of this study is its limitation, this is the first large cohort study in Thailand that provides valuable information on the real clinical impact of HLA-B*15:02 PGx testing for CBZ prescription and associated CADRs in Thai patients.

## Conclusion

The selection of appropriate AEDs was influenced by pharmacogenomic testing of HLA-B*15:02 risk alleles. *HLA-B* genotyping can guide the physician in selecting CBZ/OXC and other AEDs prescriptions rationally. The number of HLA-B*15:02 screening has increased significantly in Thailand. In 85% of cases, physicians waited for HLA-B*15:02 testing to make clinical decisions on CBZ/OXC prescribing. However, physicians have administered CBZ/OXC without PGx testing in the urgent cases. Though there were no CADRs in the HLA-B*15:02 positive group, CBZ-induced SJS/TEN were reported from HLA-B*15:02 carriers who received CBZ without PGx screening before prescription. The presence of mild rash in the HLA-B*15:02 negative group indicates that other genetic biomarker such as HLA-A*31:01 and/or non-genetic variables are involved in CBZ/OXC-induced CADRs, emphasizing that CBZ/OXC prescriptions necessitate CADRs monitoring even in patient without HLA-B*15:02 risk alleles.

## Data Availability

The original contributions presented in the study are included in the article/Supplementary Material, further inquiries can be directed to the corresponding authors.
